# Exploring the Interplay between Mitochondrial DNA and Lifestyle Factors in the Pathogenesis of Psychiatric Disorders

**DOI:** 10.1155/2024/4914777

**Published:** 2024-03-20

**Authors:** Wenming Wei, Bolun Cheng, Yijing Zhao, Dan He, Xiaoge Chu, Xiaoyue Qin, Na Zhang, Sirong Shi, Qingqing Cai, Jingni Hui, Yan Wen, Huan Liu, Yumeng Jia, Feng Zhang

**Affiliations:** Key Laboratory of Trace Elements and Endemic Diseases of National Health and Family Planning Commission, School of Public Health, Health Science Center, Xi'an Jiaotong University, Xi'an, China

## Abstract

The objectives of this study were to investigate the interaction of mitochondrial DNA (mtDNA) and lifestyle factors in the development of psychiatric disorders and to gain greater insight into their pathogenesis and comorbidity. We analyzed data from approximately 150,000 individuals from the UK Biobank. Mitochondrial gene-by-environment interaction studies (mtGEIS) were performed to assess the relationships between mtDNA and psychiatric disorders, such as anxiety, depression, and self-harm. These disorders were defined using diagnostic and severity indicators derived from the General Anxiety Disorder (GAD-7) and Patient Health Questionnaire (PHQ-9). Smoking and drinking behaviors were characterized based on UK Biobank criteria. For the mtGEIS, logistic and linear regression models from PLINK 2.0 were employed, accounting for covariates like age, sex, PC1-10, Townsend Deprivation Index (TDI), and educational attainment. We also conducted sex-stratified analyses to detect any gender-specific effects. Our findings highlighted significant associations between mtDNA and three psychiatric disorders. Moreover, the interplay between mtDNA and lifestyle factors showed significant associations with psychiatric disorders (all *P* values < 0.05). Specifically, two mutant loci, T5004C (*B*_Anx_self_ = −0.0026, *B*_Dep_self_ = −0.0024, *B*_Self−harm_ = −0.0018) and G9123A (*B*_Anx_self_ = −0.0030, *B*_Dep_self_ = −0.0024, *B*_Self−harm_ = −0.0017), were found to reduce the risk of three disorders after interacting with alcohol. Sex-specific differences were also observed. In summary, the expression of mitochondrial genes could be modulated by lifestyle factors like smoking and drinking, potentially affecting psychiatric disorders. These habits might influence mitochondrial respiratory chain activity and the replication and transcriptional regulation of mitochondrial genes, culminating in changes in mitochondrial functionality and subsequently psychiatric disorders.

## 1. Introduction

Anxiety, depression, and self-harm are widespread psychiatric disorders that significantly affect global public health [1]. The lifetime cooccurrence rates between major depressive disorder (MDD) and generalized anxiety disorder are notably high, reaching almost 50% [2]. Moreover, anxiety and depression are prominent risk factors for self-harm [3], which is a deliberate behavior characterized by self-injury or self-poisoning [4]. Self-harm is often interpreted as an indication of suicidal intent [4]. Like other psychiatric conditions, the susceptibility to anxiety disorders and major depression results from a complex interplay of heritable and nonheritable factors. The heritability for anxiety and major depression is approximated at 30% to 40%. This suggests that a notable fraction of the risk can be traced back to modifiable environmental factors [5]. However, the majority of studies examining gene-by-environment interactions in psychiatric disorders have predominantly centered on nuclear chromosomes, often overlooking the potential significance of mitochondrial DNA (mtDNA).

The human mitochondrial genome, spanning 16,569 base pairs, is intricately structured, encoding 13 proteins, 22 transfer RNAs, and 2 ribosomal RNAs. Together, these components catalyze oxidative phosphorylation reactions, producing adenosine triphosphate (ATP) as the primary cellular energy source [6]. Mitochondria, the cellular powerhouses, are pivotal in numerous metabolic pathways and signal transduction processes [7]. Mitochondrial diseases, resulting from mutations in either the mitochondrial or nuclear genomes, affect more than 10 in 100,000 individuals [8]. Psychiatric symptoms frequently correlate with mitochondrial dysfunction [9], as evidenced by a study that found a significant association between heteroplasmy in mtDNA m.13514G>A and depressive symptoms in the elderly [10]. Elevated mtDNA levels have also been associated with heightened anxiety [11]. A recent study revealed that mitochondrial autophagy in neurons might underlie the primary molecular mechanism of TNF-*α*-induced depression [12] and that mitochondrial autophagy and mtDNA could be bidirectionally regulated by reactive oxygen species (ROS). Substantial evidence suggests that mitochondrial dysfunction and oxidative stress are pivotal in the development of anxiety and depression [13]. Consequently, an increasing number of researchers are focusing on mitochondria's role in psychiatric health, genetic variant susceptibility, and their interplay with environmental factors.

The susceptibility of mitochondria to environmental exposure highlights the need to investigate the effects of smoking and drinking on this organelle. These lifestyle choices are commonly explored for their implications on psychiatric disorders. Contemporary research has associated both smoking and drinking with mtDNA mutations. Specifically, smoking has been found to amplify mtDNA heteroplasmy [14] and accelerate the accumulation of mtDNA mutations [15]. Concurrently, alcohol intake has been shown to affect mtDNA mutagenesis in blood, as evidenced by a significant increase in the relative amount of 4977 bp deleted mtDNA in alcoholics compared to controls [16]. Moreover, mtDNA seems to be more vulnerable to alterations induced by alcohol than nuclear DNA [17]. Nonetheless, a comprehensive examination of the interplay between mtDNA mutations, human behaviors, and psychiatric disorders is still lacking.

In this study, utilizing a mitochondrial gene-by-environment interaction study (mtGEIS) approach, we delved into the influence of mitochondrial single nucleotide variations (SNVs) in conjunction with smoking and alcohol consumption on anxiety, depression, and self-harm. We also conducted subgroup analyses based on sex to account for potential sex-related disparities. This research is aimed at deepening our understanding of the intricate interplay between mitochondrial genetic factors and environmental influences in the development of psychiatric disorders. It also paves the way for a richer understanding of the mechanisms behind the pathogenesis and comorbidity of these disorders. Importantly, identifying intervention strategies to mitigate the harmful effects of mitochondrial SNV interactions with smoking and alcohol on psychiatric conditions has tangible implications for real-world practices.

## 2. Methods and Materials

### 2.1. UK Biobank Cohort

The UK Biobank (UKBB) study is a prospective population-based research initiative conducted among UK residents between 2006 and 2010. The study sample comprises 502,682 individuals, all of European descent, aged 40-69 years, who were recruited from 22 assessment centers across the UK [18]. Participants underwent extensive phenotyping, including physical measurements, comprehensive health and lifestyle questionnaires, and biological sample collection. DNA was extracted from the buffy coat aliquot using the Promega Maxwell 16 Blood DNA Purification Kit (AS1010). Samples with satisfactory DNA concentration and purity, as assessed by a 260 : 280 ratio, were divided into aliquots, and 50 *μ*l of each sample was sent to Affymetrix for genotyping. The majority of participants were genotyped using the Affymetrix Axiom array (UKBB), which is a customized genotyping array with 845,485 probesets for assessing 820,967 SNVs and short insertions/deletions, including 265 mtSNVs. Further details on genotyping, imputation, quality control, and physical measurements can be found in a previous publication [19]. Our research has been approved by the UK Biobank (application 46478), which has received support from the North West Multicenter Research Ethics Committee (MREC) and the Human Tissue Authority (HTA). All participants provided informed consent for the use of their anonymous data and samples for health-related studies and the opportunity to participate in further substudies.

### 2.2. Phenotype Definition of Psychiatric Disorders

This study was aimed at investigating three common psychiatric disorders, including anxiety, depression, and self-harm, all of which were derived from UKBB. We used two indicators to measure anxiety and depression: diagnosis and severity. Anxiety severity was evaluated using the General Anxiety Disorder (GAD-7) questionnaire, which assesses seven anxiety-related symptoms and generates a total score ranging from 0 to 21 [20]. Depression severity was evaluated using the Patient Health Questionnaire (PHQ-9), a diagnostic tool that assesses the presence and severity of nine depression-related symptoms and generates a total score ranging from 0 to 27 [20]. To define the diagnostic criteria for anxiety and depression, we adhered to the guidelines set forth by Davis et al. [21]. This definition employed the GAD-7, the PHQ-9, and the Composite International Diagnostic Interview Short-Form (CIDI-SF) [22]. To measure the self-harm phenotype, we asked two questions: (1) “Have you deliberately harmed yourself, whether or not you meant to end your life?” and (2) “Have you contemplated harming yourself (for example, by cutting, biting, hitting yourself or taking an overdose)?” In our analysis, we treated “prefer not to answer” as missing data. Participants who answered “no” to both questions were classified as the control group, while those who answered “yes” were classified as the case group. Self-reported anxiety, self-reported depression, and self-harm were defined as binary variables (the case group and the control group), while anxiety score (GAD-7 score) and depression score (PHQ-9 score) were defined as a continuous variable.

### 2.3. Phenotype Definition of Smoking and Drinking

The smoking behavior criteria were derived from three distinct fields in the UKBB: 20116, 2887, and 3456. We allocated a code of 0 for respondents who indicated never having smoked, while for those who had, we tabulated the maximum number of cigarettes reported per day. As for drinking behavior, we ascertained ever alcohol consumption (UKBB field 20117) and the average weekly intake of various types of alcoholic drinks (UKBB fields 1568, 1578, 1588, 1598, 1608, and 5364). For respondents who declared not consuming alcohol, we assigned a value of 0 for their weekly alcohol intake. For the rest of the participants, we utilized the average amount of various types of alcohol consumed weekly.

### 2.4. Mitochondrial Gene-by-Environment Interaction Analysis

Using the logistic and linear regression models of PLINK 2.0 [23], the mtGEIS was performed to explore the interaction effects for psychiatric disorders. Logistic regression models were utilized to analyze the binary outcomes, specifically self-reported anxiety, self-reported depression, and self-harm. Linear regression models were employed to examine continuous outcomes, namely, anxiety score (GAD-7 score) and depression score (PHQ-9 score). The independent variables encompassed interactions between mtDNA and behavioral factors, specifically smoking and drinking habits, while the dependent variables included psychiatric traits such as anxiety, depression, and self-harm. Covariates adjusted for in our analyses encompassed age, sex, the first 10 principal components of population structure (PC1-10), daily cigarette consumption (omitted when evaluating mtDNA and smoking behavior interaction), weekly alcohol intake (omitted when evaluating mtDNA and drinking behavior interaction), Townsend Deprivation Index (TDI), and education score. Moreover, we explored the correlations between mtDNA and psychiatric disorders. To ensure a thorough examination of these relationships, we conducted identical analyses within distinct sex subgroups.

Several quality control measures were meticulously applied. Samples exhibiting over 10% missing genotype data were omitted. For SNP quality control, multiple criteria were enforced. Firstly, SNPs with a missing data rate above 5% were eliminated, leading to the removal of 22 SNPs. Secondly, SNPs with a minor allele frequency (MAF) below 1% were also excluded, resulting in the exclusion of 156 SNPs. SNPs significantly deviating from the Hardy-Weinberg equilibrium (*P* < 0.0001) were potential candidates for exclusion; however, our dataset did not contain any such SNPs. After applying these stringent criteria, 109 SNPs met our inclusion standards and were incorporated into subsequent analyses. The threshold for statistical significance was established at *P* < 0.05, aligning with the norms of genetic association studies.

## 3. Results

### 3.1. Population Characteristics

The study population comprised of 155,076 participants with GAD-7 score and 138,709 participants with self-reported anxiety (27,898 cases and 110,811 controls). Similarly, for depression, the study included 154,360 participants with PHQ-9 score and 157,459 participants with self-reported depression (76,672 cases and 80,787 controls). For self-harm, the study involved 156,669 participants with self-reported self-harm (23,616 cases and 133,053 controls). The basic characteristics of the study population can be found in Table 1.

### 3.2. Mitochondrial Gene-by-Environment Interaction Mutations with Main Effects

**Figure 1:**
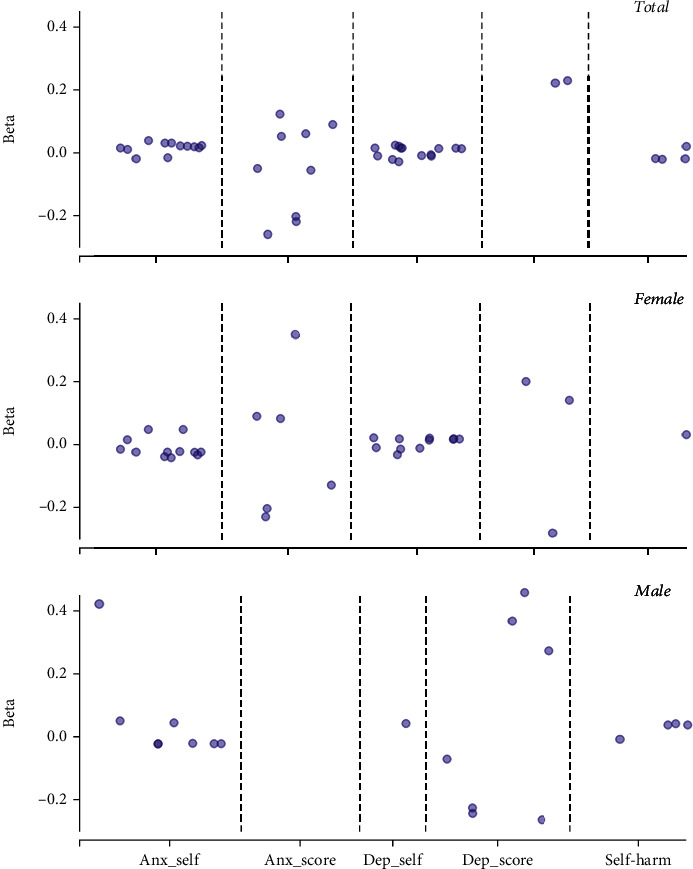
Associations of mitochondrial SNVs with psychiatric disorders. Note: only significant results were shown in this figure. Anx_self: self-reported anxiety; Anx_score: anxiety score; Dep_self: self-reported depression; Dep_score: depression score.

**Figure 2:**
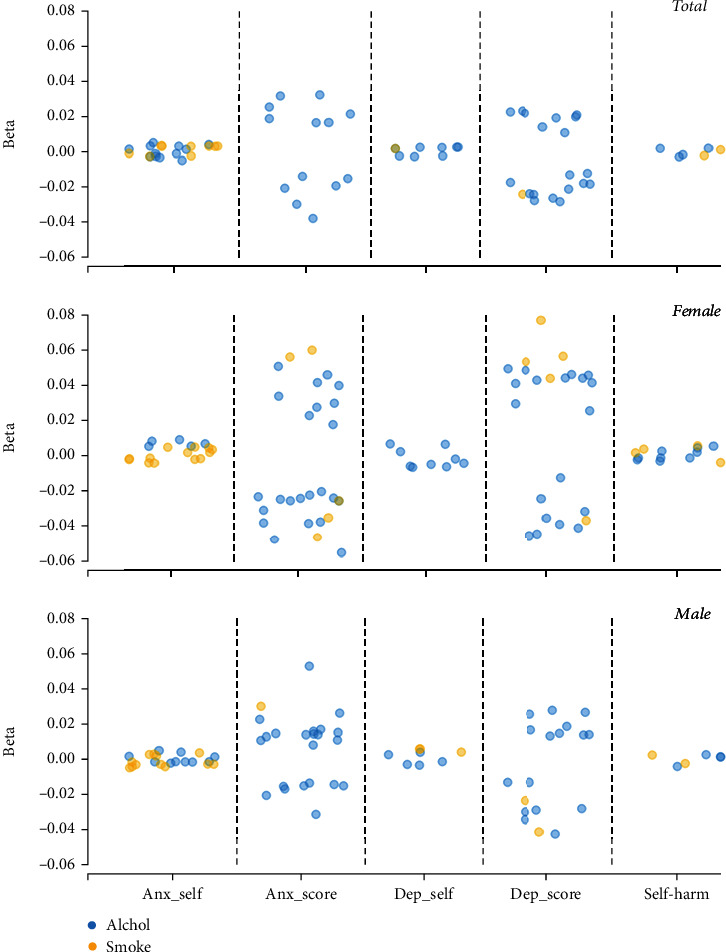
Associations of mitochondrial gene-by-environment interactions with psychiatric disorders. Note: only significant results were shown in this figure. Anx_self: self-reported anxiety; Anx_score: anxiety score; Dep_self: self-reported depression; Dep_score: depression score.

Significant correlations were found between mitochondrial SNVs and the three psychiatric disorders analyzed (all *P* values < 0.05), as depicted in Figure 1. Furthermore, there were significant correlations between the interaction of mitochondrial SNVs with smoking and drinking and the three psychiatric disorders analyzed (all *P* values < 0.05), as shown in Figure 2.

Our analysis identified significant relationships between mitochondrial SNVs and their effects on anxiety and self-harm, especially when considering the interaction with behavior. Specifically, the SNVs T9716C (*MT-CO3*) and A9667G (*MT-CO3*) showed deleterious effects on anxiety, with respective values of *B* = 0.0279 (*P* = 0.0191) and *B* = 0.2059 (*P* = 0.0487). Moreover, when accounting for alcohol interaction, the effects were further mitigating, with values of -0.0051 (*P* = 0.0005) for T9716C and -0.0380 (*P* = 0.0025) for A9667G. Conversely, the SNVs G13759A (*MT-ND5*) and T8448C (*MT-ATP8*) were associated with an aggravation in self-harm symptoms, with *B* values of 0.0221 (*P* = 0.0069) and 0.0216 (*P* = 0.0218), respectively. When combined with alcohol, these effects intensified with values of 0.0019 (*P* = 0.0408) for G13759A and 0.0030 (*P* = 0.0069) for T8448C. Please refer to Table 2 for a comprehensive summary of the results.

### 3.3. Associations of Gene-by-Environment Interactions with Risk and Severities of Psychiatric Disorders

The interaction between C3992T (*MT-ND1*) and drinking was found to have a protective effect on both the risk and severity of depression (*B*_Dep_self_ = −0.0028, *P* = 0.0075; *B*_Dep_score_ = −0.0229, *P* = 0.0232). In the male group, T10463C (*MT-TR*) was found to interact with drinking, leading to an increased risk and severity of both anxiety (*B*_Anx_self_ = 0.0015, *P* = 0.0233; *B*_Anx_score_ = 0.0157, *P* = 0.0024) and depression (*B*_Dep_self_ = 0.0014, *P* = 0.0339; *B*_Dep_score_ = 0.0148, *P* = 0.0105). In the female group, the interaction of A15924G (*MT-TT*) with smoking was found to be protective for both the risk (*B*_Anx_self_ = −0.0032, *P* = 0.0116) and severity (*B*_Anx_score_ = −0.0257, *P* = 0.0081) of anxiety, whereas the interaction with drinking was associated with increased severity of anxiety (*B*_Anx_score_ = 0.0397, *P* = 0.0002). On the other hand, in the female group, G11914A (*MT-ND4*) was found to interact with smoking, protecting against the severity of anxiety (*B*_Anx_score_ = −0.0469, *P* = 0.0086), but with drinking, increasing both the risk (*B*_Anx_self_ = 0.0052, *P* = 0.0199) and severity (*B*_Anx_score_ = 0.0415, *P* = 0.0282) of anxiety. The detailed information is shown in Tables 3 and 4.

### 3.4. Cooccurring Gene-by-Environment Interactions across Psychiatric Disorders

Two mitochondrial SNVs were found to be protective against the risk of anxiety, depression, and self-harm after interaction with drinking, including T5004C (*MT-ND2*) (*B*_Anx_self_ = −0.0026, *P* = 0.0407; *B*_Dep_self_ = −0.0024, *P* = 0.0319; *B*_Self_harm_ = −0.0018, *P* = 0.0379) and G9123A (*MT-ATP6*) (*B*_Anx_self_ = −0.0030, *P* = 0.0208; *B*_Dep_self_ = −0.0024, *P* = 0.0332; *B*_Self_harm_ = −0.0017, *P* = 0.0435). Two mitochondrial SNVs were found to be protective against anxiety and depression after interaction with drinking, including A9667G (*MT-CO3*) (*B*_Anx_score_ = −0.0380, *P* = 0.0025; *B*_Dep_score_ = −0.0264, *P* = 0.0449) and C3992T (*MT-ND1*) (*B*_Anx_self_ = −0.0029, *P* = 0.0158; *B*_Dep_self_ = −0.0028, *P* = 0.0075). SNVs that are harmful for anxiety and depression after interaction with drinking include A15924G (*MT-TT*) (*B*_Anx_score_ = 0.0152, *P* = 0.0087; *B*_Dep_score_ = 0.0124, *P* = 0.0438) and G1719A (*MT-RNR2*) (*B*_Anx_score_ = 0.0187, *P* = 0.0001; *B*_Dep_score_ = 0.0175, *P* = 0.0175). The detailed information can be found in Table 5.

### 3.5. Differences in the Effects of Smoking and Drinking Interacting with SNVs on Psychiatric Disorders

Opposing effects of drinking and smoking on three psychiatric disorders were observed after interaction with the same SNV, both in the general population and in sex subgroups. For example, C3992T (*MT-ND1*) had a protective effect on the risk of depression when interacting with drinking (*B*_Dep_score_ = −0.0229, *P* = 0.0232) but had a harmful effect on the risk of depression when interacting with smoking (*B*_Dep_score_ = 0.0241, *P* = 0.0073). The detailed information can be found in Table 4.

## 4. Discussion

Our study has demonstrated the significant impact of mtDNA on the development of psychiatric disorders, both directly and through their interaction with lifestyle elements. Furthermore, we pinpointed loci that exclusively affect psychiatric disorders through their interaction with smoking and drinking. Sex-related differences were also observed in the present study.

To our knowledge, this is the pioneering study that holistically examines the interplay between specific mitochondrial SNVs and lifestyle determinants in psychiatric conditions. We have identified the role of mitochondrial genes and their interaction with environmental factors in various psychiatric disorders. The pronounced effect of mitochondrial dysfunction on diverse body regions, especially high energy-requiring organs like the brain, is well established [24]. Subtle metabolic shifts can significantly affect neural function and increase the vulnerability to brain disorders [25], including mood disorders [26]. For instance, recent research identified a correlation between mitochondrial pathways and behaviors tied to anxiety [27]. An integrative gene set enrichment analysis spotlighted the prominence of mitochondrial-centric genes in both the bed nucleus of the stria terminalis and the blood of stress-exposed mice. Additionally, the intertwined effects of mitochondrial functionality and human behaviors on anxiety and depression risks were examined [28]. It found a positive association between mitochondrial heteroplasmy against drinking and the risks of anxiety and depression. Our research resonates with these discoveries, viewing them through the lens of mitochondrial genetics. Additionally, we have highlighted certain functional mtDNA for deeper exploration into the pathology of psychiatric conditions. Incorporating genetic screening into routine psychiatric assessments could facilitate early intervention, potentially improving outcomes by delaying or preventing the onset of disorders.

Our study suggests a role of drinking in the relationship between mtDNA and psychiatric disorders. Ethanol primarily targets the mitochondria negatively, resulting in increased oxidative stress that damages mtDNA and impairs mitochondrial function, leading to a vicious cycle of cellular damage [29]. Ethanol metabolism through alcohol dehydrogenase (ADH) produces cytosolic NADH, which is subsequently oxidized indirectly through mitochondrial electron transport, relying on metabolite carriers in the inner membrane [30]. The resulting acetaldehyde is predominantly oxidized by the mitochondrial low Km aldehyde dehydrogenase, generating NADH for further oxidation in the mitochondria. Both steps are reliant on mitochondrial electron transport [31]. Our study identified several SNVs that interacted with alcohol, including *MT-ND1-5*, *MT-ATP6*, and *MT-ATP8*. These SNVs were predominantly located within genes that encode subunits of the NADH dehydrogenase (complex I), which has been demonstrated to be the primary site of free radical generation in the electron transport chain [32]. Therefore, we hypothesize that the interaction of alcohol with mitochondria would act on mitochondrial electron transport, subsequently influencing psychiatric disorders.

Our study suggests a role of smoking in the relationship between mtDNA and psychiatric disorders. Nicotine, a primary constituent of cigarettes, permeates the central nervous system and triggers nicotinic acetylcholine receptors (nAChRs) distributed throughout the body [33]. This interaction between nicotine and nAChRs affects the mitochondrial dynamics of hippocampal neurons [34]. Previous studies have shown that nicotine-induced activation of hypoxia-inducible factor- (HIF-) 1*α* depends on mitochondria-derived ROS activation downstream of the Akt and MAPK signaling pathways, along with transcriptional regulation [35]. Our study pinpointed three SNVs that interacted with smoking, including *MT-ND1*, *MT-DLOOP*, and *MT-TT*. *MT-ND1* encodes the ND1 protein, a subunit of NADH dehydrogenase, which is located in the inner mitochondrial membrane and is the largest of the five complexes of the electron transport chain. The D-loop region is a noncoding control region in the mitochondrial genome that is essential for replication and transcriptional regulation, and alterations in this region may be associated with impaired mitochondrial biomass [36]. As a result, we propose that smoking could modulate psychiatric disorders by altering the replication and transcriptional mechanics of mitochondrial genes.

Our findings highlight the protective role of two mutant loci, T5004C (*MT-ND2*) and G9123A (*MT-ATP6*), against three psychiatric conditions (anxiety, depression, and self-harm) when they interact with alcohol consumption. We also identified a prominent mutation site, C3992T (*MT-ND1*), that has a significant impact on anxiety and depression. Specifically, this locus has been found to interact with two lifestyle factors, affecting the risk and severity of depression. In the male cohort, this specific locus was intrinsically linked to depressive symptoms. Patients with primary mtDNA mutations, such as G3460A (*MT-ND1*) and C9035T (*MT-ATP6*), have been reported to exhibit comorbidity with mood disorders [37]. However, limited research has been conducted on the comorbidity mechanisms of psychiatric disorders from a mitochondrial gene-by-environment interaction perspective. Our study provides support for the notion that smoking and alcohol consumption can influence mitochondrial respiratory chain activity, as well as the replication and transcriptional regulation of mitochondrial genes, ultimately resulting in altered mitochondrial function. In cases of mitochondrial dysfunction and cellular damage, mitochondrial contents may leak extracellularly to initiate natural immune responses [38]. Such reactions could be the latent instigators of enigmatic inflammation observed in psychiatric conditions [39].

We observed contrasting effects of smoking and drinking on three psychiatric disorders following interaction with the same SNV, both in the general population and in sex subgroups. Growing evidence suggests that mitochondria act as metabolic hubs that regulate various cellular processes [40]. Moreover, mtDNA variants may modulate classical cellular signaling pathways in a tissue-specific manner [41], which could account for the disparate or even opposing effects of the same mtDNA interacting with distinct environmental factors. Data analysis or experimental profiling of subbrain regions could be a critical step in elucidating our findings.

Our findings also highlighted sex-specific disparities. There is ample evidence indicating sex-linked differences in mitochondrial ATP production, enzyme activity, and reactive oxygen species production in various tissues and cell types [42]. Research has demonstrated that sex-specific DNA methylation plays a role in orchestrating the unique expression of nuclear genes that modulate mitochondrial performance [42]. Such modulations can indirectly lead to variances in mtDNA expressions between the sexes. Moreover, studies have reported that the detrimental effects of alcohol consumption and smoking on the epigenome may vary by sex [43]. These insights could elucidate the sex-specific psychiatric disorders resulting from the impact of alcohol and tobacco on mitochondrial functions.

This study has some innovations and limitations. We utilized the largest sample size to date to delve into the nexus between mitochondrial genes, lifestyle, and psychiatric disorders. Notably, our research marks the first attempt to unearth the impact of mitochondrial SNVs and environmental interactions on psychiatric disorders. However, some limitations should be noted. Firstly, mutations in mtDNA or mitochondrial dysfunction do not account for the etiology of all patients with psychiatric disorders, and the influence of modified genes in the nuclear genome should also be considered. Secondly, the reliance on self-reported data for smoking and drinking habits might introduce biases. Also, given its observational nature, establishing causal relationships between mtDNA, lifestyle factors, and psychiatric disorders remains challenging. A forward-looking endeavor might involve pinpointing brain regions where significant mitochondrial gene-by-environment intersections occur. It is also advisable for subsequent studies to undertake exhaustive probes into brain areas presenting with elevated somatic mtDNA mutations. Lastly, the study's participants were exclusively of European ancestry. It is imperative for future studies to encompass a more diverse range of ethnicities and races to enhance both the validity and relevance of their findings.

In conclusion, our study underscores the pivotal role of the interplay between mitochondrial genes and lifestyle factors in the development of psychiatric disorders. The research spotlighted specific genetic locations influenced by their synergy with smoking and alcohol consumption habits and discerned distinct gender-based variances. Our study hypothesizes that smoking and alcohol consumption can affect the activity of the mitochondrial respiratory chain, as well as the replication and transcriptional regulation of mitochondrial genes, leading to alterations in mitochondrial function and, ultimately, psychiatric health. Tailored public health initiatives and individual counseling could underscore the significance of lifestyle modifications, taking into account one's genetic susceptibility to psychiatric conditions. Further, our findings regarding mtDNA variations provide valuable insights into selecting personalized therapeutic approaches, accommodating the genetic profile of individuals. Such approaches could include adjusting medication doses or considering alternative treatments for those whose mtDNA variations affect drug metabolism in the context of environmental interactions. Additionally, our study calls for further investigation into the complex interplay between genetics and environmental factors in health research.

## Figures and Tables

**Table 1:** Characteristics of study participants from the UK Biobank.

Phenotype	Participants (case/control), *n*	Female (%), *n*	Age, mean (SD) (years)		
			Total	Female	Male
Self-reported anxiety	138709 (27898/110811)	77088 (55.58)	56.16 (7.70)	55.68 (7.63)	56.76 (7.74)
Anxiety score	155076	87604 (56.49)	55.89 (7.74)	55.40 (7.65)	56.53 (7.80)
Self-reported depression	157459 (76672/80787)	89746 (57.00)	56.11 (7.78)	55.63 (7.74)	56.73 (7.79)
Depression score	154360	87206 (56.50)	55.90 (7.74)	55.41 (7.65)	56.54 (7.80)
Self-harm	156669 (23616/133053)	88644 (56.58)	55.94 (7.74)	55.45 (7.66)	56.56 (7.80)

Note: SD: standard deviation.

**Table 2:** Mitochondrial gene-by-environment interaction mutations with main effects.

Phenotype	Group	Mutation	Gene	SNV-environment interactions				SNV effects		
				Environment	Beta	SE	*P*	Beta	SE	*P*
Anx_self	Total	T9716C	*MT-CO3*	Alcohol	-0.0051	0.0015	0.0005	0.0279	0.0119	0.0191
	Female	T9716C	*MT-CO3*	Alcohol	-0.0089	0.0028	0.0013	0.0425	0.0173	0.0143
	Male	T5999C	*MT-CO1*	Smoke	-0.0029	0.0014	0.0340	-0.0319	0.0139	0.0218
	Male	C14620T	*MT-ND6*	Smoke	-0.0027	0.0014	0.0477	-0.0311	0.0138	0.0241
	Male	A263G	*MT-DLOOP*	Smoke	0.0049	0.0020	0.0145	0.0415	0.0200	0.0380

Anx_score	Total	A9667G	*MT-CO3*	Alcohol	-0.0380	0.0126	0.0025	0.2059	0.1044	0.0487
	Female	G4580A	*MT-ND2*	Alcohol	0.0337	0.0146	0.0209	0.2062	0.0936	0.0275

Dep_score	Male	T5004C	*MT-ND2*	Alcohol	-0.0257	0.0131	0.0492	0.3084	0.1237	0.0127
	Male	A14582G	*MT-ND6*	Alcohol	-0.0278	0.0138	0.0432	0.2743	0.1304	0.0354
	Male	C3992T	*MT-ND1*	Alcohol	-0.0343	0.0122	0.0052	0.2362	0.1162	0.0421
	Male	C3992T	*MT-ND1*	Smoke	0.0236	0.0108	0.0294	0.2362	0.1162	0.0421
	Male	A4024G	*MT-ND1*	Alcohol	-0.0300	0.0133	0.0246	0.2537	0.1273	0.0464

Self-harm	Total	G13759A	*MT-ND5*	Alcohol	0.0019	0.0009	0.0408	0.0221	0.0082	0.0069
	Total	T8448C	*MT-ATP8*	Alcohol	0.0030	0.0011	0.0069	0.0216	0.0094	0.0218

Note: SE: standard error; SNV: single nucleotide variation; Anx_self: self-reported anxiety; Anx_score: anxiety score; Dep_score: depression score.

**Table 3:** Associations of gene-by-environment interactions with risk and severities of anxiety or depression.

Type	Group	Mutation	Gene	Environment	Self-report			Score		
					Beta	SE	*P*	Beta	SE	*P*
Anxiety	Female	A15924G	*MT-TT*	Smoke	-0.0032	0.0013	0.0116	-0.0257	0.0097	0.0081
	Female	G11914A	*MT-ND4*	Alcohol	0.0052	0.0022	0.0199	0.0415	0.0189	0.0282
	Male	A9667G	*MT-CO3*	Alcohol	0.0041	0.0017	0.0169	-0.0528	0.0144	0.0002
	Male	T10463C	*MT-TR*	Alcohol	0.0015	0.0006	0.0233	0.0157	0.0052	0.0024
	Male	A4917G	*MT-ND2*	Alcohol	0.0015	0.0007	0.0301	0.0155	0.0054	0.0040
	Male	G15928A	*MT-TT*	Alcohol	0.0013	0.0007	0.0430	0.0151	0.0053	0.0042
	Male	G14905A	*MT-CYB*	Alcohol	0.0014	0.0007	0.0378	0.0151	0.0053	0.0047
	Male	G8697A	*MT-ATP6*	Alcohol	0.0014	0.0007	0.0393	0.0151	0.0054	0.0048
	Male	A11812G	*MT-ND4*	Alcohol	0.0016	0.0007	0.0300	0.0168	0.0060	0.0049
	Male	A750G	*MT-RNR1*	Smoke	0.0042	0.0015	0.0054	0.0299	0.0125	0.0166

Depression	Total	C3992T	*MT-ND1*	Alcohol	-0.0028	0.0011	0.0075	-0.0229	0.0101	0.0232
	Male	T6221C	*MT-CO1*	Alcohol	0.0034	0.0015	0.0259	0.0287	0.0138	0.0376
	Male	C3992T	*MT-ND1*	Alcohol	-0.0030	0.0013	0.0275	-0.0343	0.0122	0.0052
	Male	T10463C	*MT-TR*	Alcohol	0.0014	0.0006	0.0339	0.0148	0.0058	0.0105

Note: SE: standard error.

**Table 4:** Mitochondrial SNVs interacting with various environmental variables in psychiatric disorders.

Type	Group	Mutation	Gene	Phenotype	Alcohol⁣^∗^			Smoke⁣^∗^		
					Beta	SE	*P*	Beta	SE	*P*
Anxiety	Total	C150T	*MT-DLOOP*	Anx_self	-0.0013	0.0006	0.0334	0.0011	0.0006	0.0477
	Female	A15924G	*MT-TT*	Anx_score	0.0397	0.0108	0.0002	-0.0257	0.0097	0.0081
	Female	G11914A	*MT-ND4*	Anx_score	0.0415	0.0189	0.0282	-0.0469	0.0179	0.0086
	Female	C3992T	*MT-ND1*	Anx_self	-0.0051	0.0024	0.0328	0.0043	0.0020	0.0293

Depression	Total	C3992T	*MT-ND1*	Dep_score	-0.0229	0.0101	0.0232	0.0241	0.0090	0.0073
	Male	T6221C	*MT-CO1*	Dep_self	0.0034	0.0015	0.0259	-0.0057	0.0019	0.0025
	Male	C6371T	*MT-CO1*	Dep_self	0.0040	0.0016	0.0109	-0.0060	0.0020	0.0026
	Male	C3992T	*MT-ND1*	Dep_score	-0.0030	0.0013	0.0275	0.0236	0.0108	0.0294

Self-harm	Female	G11914A	*MT-ND4*	Self-harm	0.0043	0.0015	0.0049	-0.0054	0.0016	0.0005

Note: SE: standard error; Anx_self: self-reported anxiety; Anx_score: anxiety score; Dep_self: self-reported depression; Dep_score: depression score. ⁣^∗^ denotes interaction with Mutation.

**Table 5:** Cooccurring gene-by-environment interactions across psychiatric disorders.

Mutation	Gene	Environment	Anxiety			Depression			Self-harm		
			Phenotype	Beta	*P*	Phenotype	Beta	*P*	Phenotype	Beta	*P*
T5004C	*MT-ND2*	Alcohol	Anx_self	-0.0026	0.0407	Dep_self	-0.0024	0.0319	Self_harm	-0.0018	0.0379
G9123A	*MT-ATP6*	Alcohol	Anx_self	-0.0030	0.0208	Dep_self	-0.0024	0.0332	Self_harm	-0.0017	0.0435
A15924G	*MT-TT*	Alcohol	Anx_score	0.0152	0.0087	Dep_score	0.0124	0.0438	—	—	—
		Smoke	—	—	—	—	—	—	Self_harm	-0.0010	0.0498
G1719A	*MT-RNR2*	Alcohol	Anx_score	0.0187	0.0001	Dep_score	0.0175	0.0005	—	—	—
C1721T	*MT-RNR2*	Alcohol	Anx_score	0.0253	0.0009	Dep_score	0.0225	0.0052	—	—	—
A9667G	*MT-CO3*	Alcohol	Anx_score	-0.0380	0.0025	Dep_score	-0.0264	0.0449	—	—	—
G16391A	*MT-DLOOP*	Alcohol	Anx_score	0.0213	0.0050	Dep_score	0.0184	0.0213	—	—	—
A4529T	*MT-ND2*	Alcohol	Anx_score	0.0207	0.0071	Dep_score	0.0218	0.0073	—	—	—
A13780G	*MT-ND5*	Alcohol	Anx_score	0.0194	0.0103	Dep_score	0.0197	0.0133	—	—	—
T10915C	*MT-ND4*	Alcohol	Anx_score	0.0322	0.0112	Dep_score	0.0284	0.0324	—	—	—
G12501A	*MT-ND5*	Alcohol	Anx_score	0.0165	0.0238	Dep_score	0.0212	0.0057	—	—	—
T10238C	*MT-ND3*	Alcohol	Anx_score	0.0164	0.0263	Dep_score	0.0191	0.0142	—	—	—
A7768G	*MT-CO2*	Alcohol	Anx_score	0.0140	0.0285	Dep_score	0.0140	0.0377	—	—	—
C3992T	*MT-ND1*	Alcohol	Anx_self	-0.0029	0.0158	Dep_self	-0.0028	0.0075	—	—	—
			—	—	—	Dep_score	-0.0229	0.0232	—	—	—
		Smoke	—	—	—	Dep_score	0.0241	0.0073	—	—	—

Note: Anx_self: self-reported anxiety; Anx_score: anxiety score; Dep_self: self-reported depression; Dep_score: depression score.

## Data Availability

The datasets generated during and/or analyzed during the current study are available from the corresponding authors upon reasonable request. Detailed analysis results are presented in the Supplementary Tables S1–S6.

## Supplementary Materials

Supplementary Materials Table S1: associations of mitochondrial SNVs with anxiety. Table S2: associations of mitochondrial SNVs with depression. Table S3: associations of mitochondrial SNVs with self-harm. Table S4: associations of mitochondrial gene-by-environment interactions with anxiety. Table S5: associations of mitochondrial gene-by-environment interactions with depression. Table S6: associations of mitochondrial gene-by-environment interactions with self-harm.
