# Supplementary material for: Exploring the Interplay between Mitochondrial DNA and Lifestyle Factors in the Pathogenesis of Psychiatric Disorders
Source: Depress Anxiety. 2024 Mar 20;2024:4914777. doi: 10.1155/2024/4914777 (PMC11918510; doi:10.1155/2024/4914777)
Supplement: Supplementary Materials — Table S1: associations of mitochondrial SNVs with anxiety. Table S2: associations of mitochondrial SNVs with depression. Table S3: associations of mitochondrial SNVs with self-harm. Table S4: associations of mitochondrial gene-by-environment interactions with anxiety. Table S5: associations of mitochondrial gene-by-environment interactions with depression. Table S6: associations of mitochondrial gene-by-environment interactions with self-harm. [file 4914777.f1.pdf]

Legends for Supplementary Tables

Table S1. Associations of Mitochondrial SNVs With Anxiety
Table S2. Associations of Mitochondrial SNVs With Depression
Table S3. Associations of Mitochondrial SNVs With Self-harm
Table S4. Associations of Mitochondrial Gene-by-environment interactions With Anxiety
Table S5. Associations of Mitochondrial Gene-by-environment interactions With Depression
Table S6. Associations of Mitochondrial Gene-by-environment interactions With Self-harm

Table S1. Associations of Mitochondrial SNVs With Anxiety

Gender	Chromosome (CHR)	SNP ID	Base Pair Position (BP)	Allele 1 (A1)	Anxiety Type	NMISS	Effect Size (BETA)	Std. Error (SE)	95% CI Lower (L95)	95% CI Upper (U95)	STAT	P-value	Gene
Total	26	Affx-92047849	4529	T	Anx_self	66868	-0.02211	0.008633	-0.03903	-0.005193	-2.562	0.01042	MT-ND2
Total	26	Affx-79381726	16391	A	Anx_self	66653	-0.0203	0.008503	-0.03697	-0.003638	-2.388	0.01695	MT-DLOOP
Total	26	Affx-34462179	6734	A	Anx_self	66680	-0.03569	0.01498	-0.06505	-0.006338	-2.383	0.01717	MT-CO1
Total	26	Affx-86886472	12501	A	Anx_self	66787	-0.01929	0.00819	-0.03534	-0.003239	-2.356	0.0185	MT-ND5
Total	26	Affx-34462338	9716	C	Anx_self	66861	0.02787	0.01189	0.004568	0.05117	2.344	0.01907	MT-CO3
Total	26	Affx-34461976	3010	A	Anx_self	66734	-0.007996	0.003412	-0.01468	-0.001309	-2.344	0.01911	MT-RNR2
Total	26	Affx-79381687	10238	C	Anx_self	66774	-0.0188	0.008294	-0.03505	-0.00254	-2.266	0.02344	MT-ND3
Total	26	Affx-79381658	1719	A	Anx_self	66769	-0.01236	0.005474	-0.02309	-0.00163	-2.258	0.02397	MT-RNR2
Total	26	Affx-79381709	15043	A	Anx_self	66741	-0.01609	0.007564	-0.03091	-0.001264	-2.127	0.03342	MT-CYB
Total	26	Affx-79381704	13780	G	Anx_self	66821	-0.01768	0.008445	-0.03424	-0.001133	-2.094	0.03625	MT-ND5
Total	26	Affx-79381694	10915	C	Anx_self	66817	-0.02791	0.01372	-0.05481	-0.001014	-2.034	0.04197	MT-ND4
Total	26	Affx-79381715	15924	G	Anx_self	66645	-0.01281	0.006342	-0.02524	-0.0003789	-2.02	0.04342	MT-TT
Total	26	Affx-34462060	4561	C	Anx_score	74657	0.2621	0.1054	0.0554	0.4687	2.485	0.01294	MT-ND2
Total	26	Affx-34462338	9716	C	Anx_score	74690	0.2223	0.09328	0.03943	0.4051	2.383	0.01719	MT-CO3
Total	26	Affx-34462182	6776	C	Anx_score	74638	-0.1204	0.05242	-0.2232	-0.01768	-2.297	0.02161	MT-CO1
Total	26	Affx-34461959	2706	A	Anx_score	74633	-0.05272	0.02373	-0.09922	-0.006216	-2.222	0.02629	MT-RNR2
Total	26	Affx-79381724	16270	T	Anx_score	74473	0.0871	0.04151	0.005748	0.1685	2.098	0.03587	MT-DLOOP
Total	26	Affx-34462190	7028	C	Anx_score	74635	-0.04932	0.02372	-0.09581	-0.002838	-2.08	0.03756	MT-CO1
Total	26	Affx-34461680	12372	A	Anx_score	74610	0.05873	0.02828	0.003298	0.1142	2.077	0.03785	MT-ND5
Total	26	Affx-79381696	11467	G	Anx_score	74536	0.05765	0.02835	0.002082	0.1132	2.033	0.04201	MT-ND4
Total	26	Affx-79381683	9667	G	Anx_score	74636	0.2059	0.1044	0.001159	0.4106	1.971	0.04872	MT-CO3
Male	26	Affx-79443437	5999	C	Anx_self	30866	-0.0319	0.0139	-0.05914	-0.00465	-2.294	0.02177	MT-CO1
Male	26	Affx-92047865	14620	T	Anx_self	30857	-0.03108	0.01378	-0.0581	-0.004067	-2.255	0.02414	MT-ND6
Male	26	Affx-79443438	6047	G	Anx_self	30877	-0.03093	0.01388	-0.05813	-0.003722	-2.228	0.02588	MT-CO1
Male	26	Affx-89025690	15693	C	Anx_self	30881	-0.0309	0.01389	-0.05813	-0.003668	-2.224	0.02615	MT-CYB
Male	26	Affx-92047873	11332	T	Anx_self	30871	-0.02968	0.01387	-0.05686	-0.002499	-2.14	0.03235	MT-ND4
Male	26	Affx-34461957	263	A	Anx_self	30810	0.04153	0.02001	0.002312	0.08075	2.076	0.03795	MT-DLOOP
Male	26	Affx-92047859	8448	C	Anx_self	30886	0.03529	0.01761	0.0007786	0.0698	2.004	0.04506	MT-ATP8
Female	26	Affx-89025745	15218	G	Anx_self	35935	0.02819	0.01102	0.006583	0.04979	2.557	0.01056	MT-CYB
Female	26	Affx-34462338	9716	C	Anx_self	35976	0.04247	0.01734	0.008489	0.07645	2.45	0.01431	MT-CO3
Female	26	Affx-79381658	1719	A	Anx_self	35931	-0.01876	0.00801	-0.03446	-0.00306	-2.342	0.01919	MT-RNR2
Female	26	Affx-34461976	3010	A	Anx_self	35921	-0.01151	0.005013	-0.02134	-0.00169	-2.297	0.02162	MT-RNR2
Female	26	Affx-79381687	10238	C	Anx_self	35944	-0.02763	0.01224	-0.05162	-0.003647	-2.258	0.02395	MT-ND3
Female	26	Affx-34461837	15758	G	Anx_self	35963	-0.03661	0.01632	-0.06859	-0.004629	-2.244	0.02486	MT-CYB
Female	26	Affx-79381694	10915	C	Anx_self	35960	-0.04572	0.02047	-0.08584	-0.005598	-2.233	0.02553	MT-ND4
Female	26	Affx-34461715	13105	G	Anx_self	35899	0.04437	0.01996	0.005247	0.0835	2.223	0.02624	MT-ND5
Female	26	Affx-79381726	16391	A	Anx_self	35863	-0.02762	0.01256	-0.05223	-0.003001	-2.199	0.02789	MT-DLOOP
Female	26	Affx-92047849	4529	T	Anx_self	35981	-0.02751	0.01276	-0.05252	-0.002497	-2.156	0.03112	MT-ND2
Female	26	Affx-86886472	12501	A	Anx_self	35940	-0.02594	0.01206	-0.04958	-0.002295	-2.15	0.03155	MT-ND5
Female	26	Affx-34462179	6734	A	Anx_self	35889	-0.04421	0.02232	-0.08796	-0.0004667	-1.981	0.04761	MT-CO1
Female	26	Affx-34462338	9716	C	Anx_score	40882	0.3459	0.1325	0.08622	0.6056	2.611	0.009039	MT-CO3
Female	26	Affx-34461959	2706	A	Anx_score	40858	-0.08637	0.03419	-0.1534	-0.01936	-2.526	0.01153	MT-RNR2
Female	26	Affx-34462060	4561	C	Anx_score	40861	0.3648	0.1488	0.07318	0.6564	2.452	0.01422	MT-ND2
Female	26	Affx-34462190	7028	C	Anx_score	40859	-0.07927	0.03418	-0.1463	-0.01228	-2.319	0.0204	MT-CO1
Female	26	Affx-79381724	16270	T	Anx_score	40777	0.133	0.05977	0.01583	0.2501	2.225	0.0261	MT-DLOOP
Female	26	Affx-34462062	4580	A	Anx_score	40768	0.2062	0.09356	0.02281	0.3895	2.204	0.02754	MT-ND2
Female	26	Affx-79381668	4336	C	Anx_score	40869	-0.2324	0.109	-0.446	-0.0188	-2.132	0.03297	MT-TQ

Note: Anx_self, self-reported anxiety; Anx_score, anxiety score

Table S2. Associations of Mitochondrial SNVs With Depression

Gender	Chromosome (CHR)	SNP ID	Base Pair Position (BP)	Allele 1 (A1)	Depression Type	NMISS	Effect Size (BETA)	Std. Error (SE)	95% CI Lower (L95)	95% CI Upper (U95)	STAT	P-value	Gene
Total	26	Affx-34461653	11914	A	Dep_score	74223	0.2254	0.09336	0.04245	0.4084	2.415	0.01575	MT-ND4
Total	26	Affx-34462338	9716	C	Dep_score	74412	0.2178	0.09874	0.02424	0.4113	2.205	0.02743	MT-CO3
Total	26	Affx-34462196	709	A	Dep_self	107510	0.01278	0.004123	0.0047	0.02086	3.1	0.001937	MT-RNR1
Total	26	Affx-79381691	10463	C	Dep_self	107739	0.01375	0.00474	0.004459	0.02304	2.901	0.003723	MT-TR
Total	26	Affx-89025732	8697	A	Dep_self	107907	0.01216	0.004865	0.00262	0.02169	2.498	0.01248	MT-ATP6
Total	26	Affx-79381671	4917	G	Dep_self	107905	0.01196	0.004874	0.002406	0.02151	2.454	0.01415	MT-ND2
Total	26	Affx-79381690	10398	G	Dep_self	107524	-0.008843	0.003609	-0.01592	-0.001769	-2.45	0.01428	MT-ND3
Total	26	Affx-34462060	4561	C	Dep_self	107972	0.0317	0.01294	0.006337	0.05707	2.45	0.0143	MT-ND2
Total	26	Affx-34461806	14905	A	Dep_self	107914	0.01149	0.004846	0.00199	0.02099	2.371	0.01776	MT-CYB
Total	26	Affx-79381664	3915	A	Dep_self	107971	0.0213	0.009004	0.003657	0.03895	2.366	0.01798	MT-ND1
Total	26	Affx-34462062	4580	A	Dep_self	107694	0.018	0.008104	0.002119	0.03389	2.221	0.02632	MT-ND2
Total	26	Affx-79381716	15928	A	Dep_self	107440	0.0102	0.004862	0.0006753	0.01973	2.099	0.03583	MT-TT
Total	26	Affx-52321592	228	A	Dep_self	107736	-0.01228	0.005977	-0.024	-0.0005671	-2.055	0.0399	MT-DLOOP
Total	26	Affx-34461648	11812	G	Dep_self	107966	0.01067	0.005357	0.000171	0.02117	1.992	0.04639	MT-ND4
Total	26	Affx-34461996	3394	C	Dep_self	107888	-0.02445	0.01235	-0.04866	-0.0002346	-1.979	0.04782	MT-ND1
Total	26	Affx-89025778	5147	A	Dep_self	107959	0.01226	0.006211	8.28E-05	0.02443	1.973	0.04846	MT-ND2
Male	26	Affx-34461653	11914	A	Dep_score	33604	0.4495	0.1307	0.1932	0.7057	3.438	0.0005866	MT-ND4
Male	26	Affx-79381672	5004	C	Dep_score	33675	0.3084	0.1237	0.06598	0.5508	2.493	0.01266	MT-ND2
Male	26	Affx-79504644	73	A	Dep_score	33642	-0.08006	0.03587	-0.1504	-0.00975	-2.232	0.02564	MT-DLOOP
Male	26	Affx-79381708	14582	G	Dep_score	33316	0.2743	0.1304	0.01877	0.5298	2.104	0.03539	MT-ND6
Male	26	Affx-79381714	15833	T	Dep_score	33647	0.2641	0.1276	0.01394	0.5143	2.069	0.03854	MT-CYB
Male	26	Affx-34461570	10044	G	Dep_score	33682	0.359	0.1737	0.01858	0.6994	2.067	0.03875	MT-TG
Male	26	Affx-79381667	3992	T	Dep_score	33666	0.2362	0.1162	0.008458	0.464	2.033	0.04209	MT-ND1
Male	26	Affx-34462030	4024	G	Dep_score	33671	0.2537	0.1273	0.004098	0.5033	1.992	0.04636	MT-ND1
Male	26	Affx-79381664	3915	A	Dep_self	46825	0.03283	0.01326	0.006842	0.05881	2.476	0.01329	MT-ND1
Female	26	Affx-34462338	9716	C	Dep_score	40723	0.2876	0.1353	0.02244	0.5527	2.126	0.03352	MT-CO3
Female	26	Affx-34462062	4580	A	Dep_score	40610	0.1974	0.09566	0.009891	0.3849	2.063	0.03909	MT-ND2
Female	26	Affx-79381700	12705	T	Dep_score	40693	-0.1346	0.06693	-0.2657	-0.003381	-2.01	0.04439	MT-ND5
Female	26	Affx-79381691	10463	C	Dep_self	61038	0.01684	0.006389	0.004318	0.02936	2.636	0.008395	MT-TR
Female	26	Affx-34462196	709	A	Dep_self	60899	0.01371	0.005554	0.002822	0.02459	2.468	0.01359	MT-RNR1
Female	26	Affx-34461803	14798	C	Dep_self	61153	-0.01293	0.005333	-0.02338	-0.002477	-2.424	0.01533	MT-CYB
Female	26	Affx-34461806	14905	A	Dep_self	61127	0.01519	0.006518	0.002419	0.02797	2.331	0.01976	MT-CYB
Female	26	Affx-89025732	8697	A	Dep_self	61103	0.0152	0.00655	0.002365	0.02804	2.321	0.02029	MT-ATP6
Female	26	Affx-52321592	228	A	Dep_self	61033	-0.01812	0.008021	-0.03384	-0.002397	-2.259	0.0239	MT-DLOOP
Female	26	Affx-79381671	4917	G	Dep_self	61111	0.01433	0.006556	0.001482	0.02718	2.186	0.02882	MT-ND2
Female	26	Affx-79381690	10398	G	Dep_self	60901	-0.01052	0.004863	-0.02005	-0.0009856	-2.163	0.03057	MT-ND3
Female	26	Affx-79381716	15928	A	Dep_self	60849	0.01411	0.006541	0.001286	0.02693	2.157	0.03104	MT-TT
Female	26	Affx-89025778	5147	A	Dep_self	61154	0.01785	0.008331	0.00152	0.03418	2.142	0.03216	MT-ND2
Female	26	Affx-34462060	4561	C	Dep_self	61147	0.03613	0.01737	0.002074	0.07018	2.079	0.03759	MT-ND2

Note: Dep_self, self-reported depression; Dep_score, depression score

Table S3. Associations of Mitochondrial SNVs With Self-harm

Gender	Chromosome (CHR)	SNP ID	Base Pair Position (BP)	Allele 1 (A1)	NMISS	Effect Size (BETA)	Std. Error (SE)	95% CI Lower (L95)	95% CI Upper (U95)	STAT	P-value	Gene
Total	26	Affx-79443499	13759	A	108280	0.02206	0.008159	0.006071	0.03805	2.704	0.006852	MT-ND5
Total	26	Affx-79381683	9667	G	108610	-0.02361	0.009465	-0.04216	-0.005062	-2.495	0.01261	MT-CO3
Total	26	Affx-92047859	8448	C	108702	0.02162	0.009426	0.003141	0.04009	2.293	0.02184	MT-ATP8
Total	26	Affx-34461763	13966	G	108639	0.0171	0.007773	0.001865	0.03233	2.2	0.02782	MT-ND5
Male	26	Affx-34461653	11914	A	47352	0.02843	0.0106	0.007657	0.04921	2.682	0.007313	MT-ND4
Male	26	Affx-34461715	13105	G	47364	0.03277	0.01348	0.00636	0.05919	2.432	0.01502	MT-ND5
Male	26	Affx-34462062	4580	A	47340	-0.01695	0.008085	-0.0328	-0.001107	-2.097	0.03601	MT-ND2
Male	26	Affx-92047866	14869	A	45283	0.02844	0.0144	0.0002221	0.05665	1.975	0.04823	MT-CYB
Female	26	Affx-79443499	13759	A	61005	0.02455	0.0115	0.002009	0.0471	2.135	0.0328	MT-ND5

Table S4. Associations of Mitochondrial Gene-by-environment interactions With Anxiety

Gender	Chromosome (CHR)	SNP ID	Base Pair Position (BP)	Allele 1 (A1)	Anxiety Type	Environment	NMISS	Effect Size (BETA)	Std. Error (SE)	95% CI Lower (L95)	95% CI Upper (U95)	STAT	P-value	Gene
Total	26	Affx-79381658	1719	A	Anx score	alcoholfreqweek	74586	0.0187	0.004734	0.009417	0.02797	3.949	7.85E-05	MT-RNR2
Total	26	Affx-79443409	1721	T	Anx score	alcoholfreqweek	74697	0.02525	0.007623	0.01031	0.04019	3.313	0.0009247	MT-RNR2
Total	26	Affx-34462338	9716	C	Anx self	alcoholfreqweek	66861	-0.00509	0.00146	-0.007952	-0.002228	-3.486	0.00049	MT-CO3
Total	26	Affx-79381683	9667	G	Anx score	alcoholfreqweek	74636	-0.03798	0.01257	-0.06262	-0.01335	-3.022	0.002513	MT-CO3
Total	26	Affx-34462060	4561	C	Anx self	alcoholfreqweek	66829	-0.005043	0.00167	-0.008316	-0.00177	-3.02	0.002529	MT-ND2
Total	26	Affx-79381708	14582	G	Anx self	alcoholfreqweek	66068	-0.003968	0.001363	-0.006638	-0.001297	-2.912	0.003598	MT-ND6
Total	26	Affx-79381667	3992	T	Anx self	alcoholfreqweek	66828	-0.002901	0.001202	-0.005256	-0.0005453	-2.414	0.01579	MT-ND1
Total	26	Affx-34462030	4024	G	Anx self	alcoholfreqweek	66840	-0.003137	0.00132	-0.005724	-0.0005504	-2.377	0.01746	MT-ND1
Total	26	Affx-79381726	16391	A	Anx score	alcoholfreqweek	74454	0.02133	0.007596	0.006446	0.03622	2.809	0.004978	MT-DLOOP
Total	26	Affx-92047849	4529	T	Anx score	alcoholfreqweek	74694	0.02074	0.007709	0.005628	0.03585	2.69	0.007145	MT-ND2
Total	26	Affx-79381715	15924	G	Anx score	alcoholfreqweek	74449	0.01524	0.005808	0.003853	0.02662	2.623	0.008709	MT-TT
Total	26	Affx-79381704	13780	G	Anx score	alcoholfreqweek	74642	0.01935	0.007544	0.004567	0.03414	2.565	0.01031	MT-ND5
Total	26	Affx-79381694	10915	C	Anx score	alcoholfreqweek	74645	0.0322	0.01269	0.007321	0.05708	2.537	0.01119	MT-ND4
Total	26	Affx-92047864	9123	A	Anx self	alcoholfreqweek	66817	-0.003017	0.001306	-0.005576	-0.0004584	-2.311	0.02083	MT-ATP6
Total	26	Affx-79381691	10463	C	Anx self	alcoholfreqweek	66685	0.001268	0.0005658	0.0001592	0.002377	2.241	0.02501	MT-TR
Total	26	Affx-34462122	5656	G	Anx self	alcoholfreqweek	66842	-0.003413	0.001575	-0.0065	-0.0003264	-2.167	0.03023	
Total	26	Affx-52321525	150	T	Anx self	alcoholfreqweek	66759	-0.001292	0.0006071	-0.002482	-0.000102	-2.128	0.03335	MT-DLOOP
Total	26	Affx-89025732	8697	A	Anx self	alcoholfreqweek	66788	0.001214	0.000585	6.70E-05	0.00236	2.074	0.03804	MT-ATP6
Total	26	Affx-79381672	5004	C	Anx self	alcoholfreqweek	66834	-0.002635	0.001288	-0.005159	-0.0001115	-2.047	0.04071	MT-ND2
Total	26	Affx-79381671	4917	G	Anx self	alcoholfreqweek	66789	0.001172	0.0005872	2.06E-05	0.002322	1.995	0.04604	MT-ND2
Total	26	Affx-79443437	5999	C	Anx self	smokefreqday	66819	-0.003458	0.001259	-0.005925	-0.0009909	-2.747	0.006011	MT-CO1
Total	26	Affx-79381664	3915	A	Anx self	smokefreqday	66819	0.002771	0.001012	0.0007875	0.004755	2.738	0.006181	MT-ND1
Total	26	Affx-92047865	14620	T	Anx self	smokefreqday	66816	-0.003	0.001249	-0.005447	-0.0005522	-2.402	0.0163	MT-ND6
Total	26	Affx-79443438	6047	G	Anx self	smokefreqday	66844	-0.002926	0.001266	-0.005408	-0.0004435	-2.31	0.02088	MT-CO1
Total	26	Affx-92047873	11332	T	Anx self	smokefreqday	66841	-0.002887	0.001264	-0.005365	-0.0004091	-2.284	0.0224	MT-ND4
Total	26	Affx-89025690	15693	C	Anx self	smokefreqday	66869	-0.002878	0.001266	-0.005359	-0.0003957	-2.272	0.02306	MT-CYB
Total	26	Affx-79381695	11377	A	Anx self	smokefreqday	66722	0.002435	0.001177	0.0001276	0.004743	2.068	0.03862	MT-ND4
Total	26	Affx-89025703	16193	T	Anx self	smokefreqday	66667	0.00302	0.001472	0.0001342	0.005906	2.051	0.04026	MT-DLOOP
Total	26	Affx-52321525	150	T	Anx self	smokefreqday	66759	0.001118	0.0005644	1.15E-05	0.002224	1.98	0.04767	MT-DLOOP
Total	26	Affx-86886472	12501	A	Anx score	alcoholfreqweek	74611	0.01646	0.007279	0.00219	0.03072	2.261	0.02378	MT-ND5
Total	26	Affx-79381687	10238	C	Anx score	alcoholfreqweek	74589	0.01638	0.007372	0.001935	0.03083	2.222	0.02626	MT-ND3
Total	26	Affx-79381663	3720	G	Anx score	alcoholfreqweek	74593	0.03155	0.01437	0.003383	0.05972	2.195	0.02814	MT-ND1
Total	26	Affx-79381679	7768	G	Anx score	alcoholfreqweek	74686	0.01396	0.006375	0.001467	0.02646	2.19	0.02852	MT-CO2
Total	26	Affx-34462179	6734	A	Anx score	alcoholfreqweek	74479	0.02987	0.0139	0.002632	0.05712	2.149	0.03161	MT-CO1
Male	26	Affx-79381683	9667	G	Anx score	alcoholfreqweek	33788	-0.05283	0.01438	-0.08102	-0.02465	-3.674	0.0002393	MT-CO3
Male	26	Affx-89025745	15218	G	Anx score	alcoholfreqweek	33767	-0.02605	0.00845	-0.04261	-0.009484	-3.082	0.002055	MT-CYB
Male	26	Affx-79381691	10463	C	Anx score	alcoholfreqweek	33709	0.01574	0.005182	0.005587	0.0259	3.038	0.002382	MT-TR
Male	26	Affx-89025674	497	T	Anx score	alcoholfreqweek	33792	-0.02245	0.007752	-0.03764	-0.007256	-2.896	0.003782	MT-DLOOP
Male	26	Affx-79381671	4917	G	Anx score	alcoholfreqweek	33758	0.0155	0.005384	0.004951	0.02606	2.88	0.003984	MT-ND2
Male	26	Affx-79381716	15928	A	Anx score	alcoholfreqweek	33621	0.01514	0.005285	0.004782	0.0255	2.865	0.004175	MT-TT
Male	26	Affx-34461806	14905	A	Anx score	alcoholfreqweek	33760	0.01507	0.005326	0.004632	0.02551	2.83	0.004663	MT-CYB
Male	26	Affx-89025732	8697	A	Anx score	alcoholfreqweek	33777	0.0151	0.005351	0.004609	0.02559	2.821	0.004786	MT-ATP6
Male	26	Affx-34461648	11812	G	Anx score	alcoholfreqweek	33792	0.01675	0.005958	0.005073	0.02843	2.811	0.004936	MT-ND4
Male	26	Affx-79381661	3480	G	Anx score	alcoholfreqweek	33796	-0.01461	0.005813	-0.02601	-0.003218	-2.514	0.01196	MT-ND1
Male	26	Affx-89025746	14167	T	Anx score	alcoholfreqweek	33792	-0.01437	0.005837	-0.02581	-0.00293	-2.462	0.01382	MT-ND6
Male	26	Affx-34461803	14798	C	Anx score	alcoholfreqweek	33790	-0.01085	0.004426	-0.01952	-0.002176	-2.452	0.01422	MT-CYB
Male	26	Affx-89025778	5147	A	Anx score	alcoholfreqweek	33776	0.01693	0.006923	0.003364	0.0305	2.446	0.01446	MT-ND2
Male	26	Affx-34461593	10550	G	Anx score	alcoholfreqweek	33798	-0.0142	0.005831	-0.02563	-0.002773	-2.435	0.01488	MT-ND4L
Male	26	Affx-34462196	709	A	Anx score	alcoholfreqweek	33661	0.01068	0.004416	0.002028	0.01934	2.419	0.01556	MT-RNR1
Male	26	Affx-89025685	9055	A	Anx score	alcoholfreqweek	33742	-0.01386	0.005729	-0.02509	-0.002631	-2.419	0.01556	MT-ATP6
Male	26	Affx-89025753	11299	C	Anx score	alcoholfreqweek	33803	-0.01394	0.005817	-0.02534	-0.002537	-2.396	0.01658	MT-ND4
Male	26	Affx-79381656	750	A	Anx score	smokefreqday	33084	0.0299	0.01248	0.005432	0.05437	2.395	0.01662	MT-RNR1
Male	26	Affx-79443409	1721	T	Anx score	alcoholfreqweek	33806	0.02063	0.008632	0.003709	0.03755	2.39	0.01687	MT-RNR2
Male	26	Affx-79381658	1719	A	Anx score	alcoholfreqweek	33758	0.01264	0.005476	0.001912	0.02338	2.309	0.02094	MT-RNR2
Male	26	Affx-79381684	9698	C	Anx score	alcoholfreqweek	33706	-0.01353	0.005861	-0.02501	-0.00204	-2.308	0.02101	MT-CO3
Male	26	Affx-79381694	10915	C	Anx score	alcoholfreqweek	33780	0.03143	0.01477	0.002476	0.06038	2.128	0.03338	MT-ND4
Male	26	Affx-79381690	10398	G	Anx score	alcoholfreqweek	33634	-0.008064	0.003972	-0.01585	-0.000279	-2.03	0.04234	MT-ND3
Male	26	Affx-34462196	709	A	Anx self	smokefreqday	30751	-0.001596	0.0005157	-0.002606	-0.0005849	-3.094	0.001976	MT-RNR1
Male	26	Affx-79381656	750	A	Anx self	smokefreqday	30213	0.004243	0.001525	0.001254	0.007232	2.782	0.005401	MT-RNR1
Male	26	Affx-34461788	1438	A	Anx self	smokefreqday	30832	0.003108	0.001182	0.000791	0.005425	2.629	0.008565	MT-RNR1
Male	26	Affx-34462122	5656	G	Anx self	alcoholfreqweek	30869	-0.004908	0.001872	-0.008578	-0.001238	-2.621	0.00876	
Male	26	Affx-34461957	263	A	Anx self	smokefreqday	30810	0.004896	0.002003	0.0009699	0.008822	2.444	0.01452	MT-DLOOP
Male	26	Affx-34462075	4769	A	Anx self	smokefreqday	30871	0.002964	0.001221	0.0005711	0.005356	2.428	0.01519	MT-ND2
Male	26	Affx-79381683	9667	G	Anx self	alcoholfreqweek	30867	0.00405	0.001695	0.0007277	0.007372	2.389	0.01689	MT-CO3
Male	26	Affx-79381664	3915	A	Anx self	smokefreqday	30863	0.002716	0.001147	0.0004681	0.004965	2.368	0.01789	MT-ND1
Male	26	Affx-34462179	6734	A	Anx self	smokefreqday	30791	0.004253	0.001821	0.0006833	0.007822	2.335	0.01954	MT-CO1
Male	26	Affx-52321525	150	T	Anx self	alcoholfreqweek	30844	-0.001643	0.000705	-0.003025	-0.0002614	-2.331	0.01977	MT-DLOOP
Male	26	Affx-79381691	10463	C	Anx self	alcoholfreqweek	30787	0.001456	0.0006414	0.0001985	0.002713	2.269	0.02325	MT-TR
Male	26	Affx-89025778	5147	A	Anx self	smokefreqday	30852	-0.001852	0.0008167	-0.003452	-0.0002511	-2.267	0.02337	MT-ND2
Male	26	Affx-79381679	7768	G	Anx self	alcoholfreqweek	30882	-0.002255	0.001005	-0.004226	-0.0002847	-2.243	0.0249	MT-CO2
Male	26	Affx-34461648	11812	G	Anx self	alcoholfreqweek	30872	0.001619	0.0007463	0.0001566	0.003082	2.17	0.03003	MT-ND4
Male	26	Affx-79381671	4917	G	Anx self	alcoholfreqweek	30840	0.001453	0.00067	0.0001398	0.002766	2.169	0.03012	MT-ND2
Male	26	Affx-34461715	13105	G	Anx self	smokefreqday	30814	-0.003663	0.001711	-0.007016	-0.0003106	-2.142	0.03224	MT-ND5
Male	26	Affx-79443437	5999	C	Anx self	smokefreqday	30866	-0.002938	0.001386	-0.005655	-0.0002218	-2.12	0.03402	MT-CO1
Male	26	Affx-34461806	14905	A	Anx self	alcoholfreqweek	30845	0.001377	0.0006627	7.80E-05	0.002676	2.078	0.03776	MT-CYB
Male	26	Affx-89025732	8697	A	Anx self	alcoholfreqweek	30857	0.001371	0.0006652	6.76E-05	0.002675	2.062	0.03925	MT-ATP6
Male	26	Affx-79381716	15928	A	Anx self	alcoholfreqweek	30721	0.001329	0.0006566	4.19E-05	0.002616	2.024	0.04301	MT-TT
Male	26	Affx-34461837	15758	G	Anx self	smokefreqday	30868	-0.002812	0.001397	-0.00555	-7.44E-05	-2.013	0.0441	MT-CYB
Male	26	Affx-92047865	14620	T	Anx self	smokefreqday	30857	-0.002723	0.001375	-0.005418	-2.81E-05	-1.98	0.04767	MT-ND6
Female	26	Affx-79381726	16391	A	Anx score	alcoholfreqweek	40747	0.05589	0.01453	0.02741	0.08437	3.846	0.00012	MT-DLOOP
Female	26	Affx-79381715	15924	G	Anx score	alcoholfreqweek	40748	0.03974	0.01077	0.01864	0.06084	3.691	0.0002237	MT-TT
Female	26	Affx-79381690	10398	G	Anx score	alcoholfreqweek	40684	0.02267	0.006236	0.01045	0.0349	3.636	0.0002771	MT-ND3
Female	26	Affx-79381658	1719	A	Anx score	alcoholfreqweek	40828	0.03111	0.008752	0.01396	0.04826	3.554	0.0003792	MT-RNR2
Female	26	Affx-92047849	4529	T	Anx score	alcoholfreqweek	40883	0.05069	0.0148	0.02169	0.07969	3.426	0.0006139	MT-ND2
Female	26	Affx-79381704	13780	G	Anx score	alcoholfreqweek	40860	0.0459	0.01435	0.01778	0.07402	3.199	0.001381	MT-ND5
Female	26	Affx-34462196	709	A	Anx score	alcoholfreqweek	40688	-0.02329	0.007372	-0.03774	-0.008843	-3.159	0.001582	MT-RNR1
Female	26	Affx-79381716	15928	A	Anx score	alcoholfreqweek	40681	-0.02577	0.008714	-0.04284	-0.008687	-2.957	0.003109	MT-TT
Female	26	Affx-34461648	11812	G	Anx score	alcoholfreqweek	40857	-0.02735	0.009549	-0.04607	-0.008639	-2.865	0.004177	MT-ND4
Female	26	Affx-79381671	4917	G	Anx score	alcoholfreqweek	40847	-0.02481	0.008748	-0.04195	-0.007661	-2.836	0.004574	MT-ND2
Female	26	Affx-79381687	10238	C	Anx score	alcoholfreqweek	40840	0.03862	0.01374	0.0117	0.06555	2.811	0.004934	MT-ND3
Female	26	Affx-79381694	10915	C	Anx score	smokefreqday	40865	-0.05995	0.02146	-0.102	-0.01789	-2.793	0.005218	MT-ND4
Female	26	Affx-86886472	12501	A	Anx score	alcoholfreqweek	40840	0.03787	0.01362	0.01117	0.06458	2.78	0.005442	MT-ND5
Female	26	Affx-34461806	14905	A	Anx score	alcoholfreqweek	40841	-0.02412	0.008686	-0.04114	-0.007091	-2.776	0.005501	MT-CYB
Female	26	Affx-89025732	8697	A	Anx score	alcoholfreqweek	40835	-0.02429	0.008805	-0.04154	-0.007027	-2.758	0.005818	MT-ATP6
Female	26	Affx-79443409	1721	T	Anx score	alcoholfreqweek	40891	0.03835	0.01443	0.01007	0.06663	2.658	0.007871	MT-RNR2
Female	26	Affx-79381715	15924	G	Anx score	smokefreqday	40748	-0.02565	0.009689	-0.04464	-0.006659	-2.647	0.008118	MT-TT
Female	26	Affx-34461653	11914	A	Anx score	smokefreqday	40779	-0.04689	0.01785	-0.08187	-0.0119	-2.626	0.008632	MT-ND4
Female	26	Affx-79381691	10463	C	Anx score	alcoholfreqweek	40794	-0.02237	0.008543	-0.03912	-0.005631	-2.619	0.00882	MT-TR
Female	26	Affx-34461803	14798	C	Anx score	alcoholfreqweek	40863	0.01752	0.006831	0.004129	0.03091	2.564	0.01034	MT-CYB
Female	26	Affx-34462062	4580	A	Anx score	alcoholfreqweek	40768	0.03367	0.01457	0.005107	0.06223	2.31	0.02087	MT-ND2
Female	26	Affx-34462182	6776	C	Anx score	alcoholfreqweek	40864	0.02565	0.01114	0.003819	0.04749	2.303	0.0213	MT-CO1
Female	26	Affx-79381709	15043	A	Anx score	alcoholfreqweek	40799	0.02971	0.01313	0.003981	0.05544	2.263	0.02362	MT-CYB
Female	26	Affx-34462179	6734	A	Anx score	smokefreqday	40771	-0.05609	0.02501	-0.1051	-0.00708	-2.243	0.0249	MT-CO1
Female	26	Affx-34461763	13966	G	Anx score	smokefreqday	40870	0.03538	0.01586	0.004296	0.06646	2.231	0.0257	MT-ND5
Female	26	Affx-34461653	11914	A	Anx score	alcoholfreqweek	40779	0.04146	0.01889	0.004432	0.07848	2.195	0.0282	MT-ND4
Female	26	Affx-34462338	9716	C	Anx self	alcoholfreqweek	35976	-0.008923	0.002775	-0.01436	-0.003485	-3.216	0.001301	MT-CO3
Female	26	Affx-89025739	3796	G	Anx score	alcoholfreqweek	40866	0.04811	0.02292	0.003186	0.09303	2.099	0.03582	MT-ND1
Female	26	Affx-79381700	12705	T	Anx score	alcoholfreqweek	40850	0.02039	0.009847	0.001087	0.03969	2.07	0.03843	MT-ND5
Female	26	Affx-34462060	4561	C	Anx self	alcoholfreqweek	35957	-0.008047	0.003156	-0.01423	-0.001862	-2.55	0.01078	MT-ND2
Female	26	Affx-79381715	15924	G	Anx self	smokefreqday	35858	-0.003189	0.001264	-0.005666	-0.000713	-2.524	0.0116	MT-TT
Female	26	Affx-79381708	14582	G	Anx self	alcoholfreqweek	35533	-0.006578	0.002711	-0.01189	-0.001263	-2.426	0.01527	MT-ND6
Female	26	Affx-34461691	12612	G	Anx self	smokefreqday	35916	0.002287	0.0009609	0.0004042	0.004171	2.381	0.01729	MT-ND5
Female	26	Affx-34461653	11914	A	Anx self	alcoholfreqweek	35889	0.005179	0.002224	0.0008201	0.009537	2.329	0.01988	MT-ND4
Female	26	Affx-34461828	15452	A	Anx self	smokefreqday	35820	0.001671	0.0007313	0.0002381	0.003105	2.285	0.02229	MT-CYB
Female	26	Affx-89025684	4216	C	Anx self	smokefreqday	35951	0.001639	0.0007274	0.0002137	0.003065	2.254	0.02422	MT-ND1
Female	26	Affx-79381667	3992	T	Anx self	smokefreqday	35961	0.004304	0.001974	0.0004337	0.008173	2.18	0.02929	MT-ND1
Female	26	Affx-34461745	13708	A	Anx self	smokefreqday	35846	0.001915	0.0008935	0.0001632	0.003666	2.143	0.03215	MT-ND5
Female	26	Affx-79381710	15257	A	Anx self	smokefreqday	35969	0.004263	0.001994	0.0003541	0.008172	2.137	0.03256	MT-CYB
Female	26	Affx-79381667	3992	T	Anx self	alcoholfreqweek	35961	-0.00509	0.002385	-0.009764	-0.0004157	-2.134	0.03282	MT-ND1
Female	26	Affx-79443447	7476	T	Anx self	smokefreqday	35976	0.004556	0.002144	0.0003527	0.008759	2.124	0.03364	MT-TS1
Female	26	Affx-34461623	11251	G	Anx self	smokefreqday	35886	0.001529	0.000732	9.37E-05	0.002963	2.088	0.03681	MT-ND4
Female	26	Affx-89025731	12633	A	Anx self	smokefreqday	35953	-0.004672	0.002245	-0.009072	-0.0002718	-2.081	0.03744	MT-ND5
Female	26	Affx-79381672	5004	C	Anx self	smokefreqday	35965	0.004438	0.002148	0.0002291	0.008647	2.067	0.03878	MT-ND2
Female	26	Affx-52321592	228	A	Anx self	smokefreqday	35890	0.002493	0.001213	0.0001145	0.004871	2.054	0.03995	MT-DLOOP
Female	26	Affx-92047869	295	T	Anx self	smokefreqday	35713	0.001918	0.0009649	2.74E-05	0.00381	1.988	0.04679	MT-DLOOP

Note: Anx self, self-reported anxiety; Anx score, anxiety score

Table S5. Associations of Mitochondrial Gene-by-environment interactions With Depression

Gender	Chromosome (CHR)	SNP ID	Base Pair Position (BP)	Allele 1 (A1)	Depression Type	Environment	NMISS	Effect Size (BETA)	Std. Error (SE)	95% CI Lower (L95)	95% CI Upper (U95)	STAT	P-value	Gene
Total	26	Affx-79381658	1719	A	Dep_score	alcoholfreqweek	74309	0.01748	0.004989	0.007702	0.02726	3.504	0.0004588	MT-RNR2
Total	26	Affx-79443409	1721	T	Dep_score	alcoholfreqweek	74421	0.02247	0.008043	0.006711	0.03824	2.794	0.005201	MT-RNR2
Total	26	Affx-86886472	12501	A	Dep_score	alcoholfreqweek	74335	0.02119	0.007667	0.006164	0.03622	2.764	0.005712	MT-ND5
Total	26	Affx-79381667	3992	T	Dep_score	smokefreqday	74371	0.02414	0.008992	0.006515	0.04176	2.685	0.007265	MT-ND1
Total	26	Affx-92047849	4529	T	Dep_score	alcoholfreqweek	74416	0.02179	0.008122	0.005875	0.03771	2.683	0.007291	MT-ND2
Total	26	Affx-79381704	13780	G	Dep_score	alcoholfreqweek	74363	0.01967	0.007945	0.004098	0.03524	2.476	0.0133	MT-ND5
Total	26	Affx-79381687	10238	C	Dep_score	alcoholfreqweek	74314	0.01906	0.007769	0.003832	0.03429	2.453	0.01416	MT-ND3
Total	26	Affx-79381700	12705	T	Dep_score	alcoholfreqweek	74344	0.01319	0.005478	0.002453	0.02392	2.408	0.01606	MT-ND5
Total	26	Affx-89025742	6371	T	Dep_score	alcoholfreqweek	74323	0.02776	0.01159	0.005035	0.05048	2.394	0.01666	MT-CO1
Total	26	Affx-89025745	15218	G	Dep_score	alcoholfreqweek	74322	-0.01801	0.007721	-0.03315	-0.002882	-2.333	0.01964	MT-CYB
Total	26	Affx-79381726	16391	A	Dep_score	alcoholfreqweek	74177	0.01838	0.007981	0.002741	0.03402	2.303	0.02126	MT-DLOOP
Total	26	Affx-79381667	3992	T	Dep_score	alcoholfreqweek	74371	-0.02294	0.0101	-0.04275	-0.003136	-2.27	0.02319	MT-ND1
Total	26	Affx-79381678	6221	C	Dep_score	alcoholfreqweek	74353	0.02432	0.0112	0.002367	0.04628	2.171	0.02991	MT-CO1
Total	26	Affx-79381694	10915	C	Dep_score	alcoholfreqweek	74368	0.02837	0.01326	0.002384	0.05436	2.14	0.03238	MT-ND4
Total	26	Affx-79381679	7768	G	Dep_score	alcoholfreqweek	74409	0.01396	0.006716	0.0007935	0.02712	2.078	0.0377	MT-CO2
Total	26	Affx-79381715	15924	G	Dep_score	alcoholfreqweek	74171	0.01236	0.006133	0.000344	0.02438	2.016	0.0438	MT-TT
Total	26	Affx-79381683	9667	G	Dep_score	alcoholfreqweek	74360	-0.02641	0.01317	-0.05222	-0.0006009	-2.006	0.0449	MT-CO3
Total	26	Affx-34461763	13966	G	Dep_score	alcoholfreqweek	74377	0.02075	0.01038	0.0003954	0.0411	1.998	0.04572	MT-ND5
Total	26	Affx-79381676	5495	C	Dep_score	alcoholfreqweek	74320	-0.02391	0.01208	-0.04758	-0.0002447	-1.98	0.04768	MT-ND2
Total	26	Affx-34461648	11812	G	Dep_score	alcoholfreqweek	74370	0.01068	0.005407	8.12E-05	0.02128	1.975	0.04827	MT-ND4
Total	26	Affx-79381667	3992	T	Dep_self	alcoholfreqweek	107948	-0.002827	0.001057	-0.004898	-0.0007563	-2.676	0.007457	MT-ND1
Total	26	Affx-89025715	11674	T	Dep_self	alcoholfreqweek	108028	-0.002496	0.001156	-0.004762	-0.0002303	-2.159	0.03083	MT-ND4
Total	26	Affx-89025674	497	T	Dep_self	smokefreqday	107979	0.001653	0.0007663	0.0001508	0.003155	2.157	0.03103	MT-DLOOP
Total	26	Affx-79381672	5004	C	Dep_self	alcoholfreqweek	107982	-0.002397	0.001117	-0.004586	-0.0002069	-2.145	0.03194	MT-ND2
Total	26	Affx-92047864	9123	A	Dep_self	alcoholfreqweek	107952	-0.002391	0.001123	-0.004591	-0.0001904	-2.13	0.03321	MT-ATP6
Total	26	Affx-89025749	8994	A	Dep_self	alcoholfreqweek	108012	-0.002305	0.00109	-0.00444	-0.0001695	-2.116	0.03439	MT-ATP6
Total	26	Affx-34461684	1243	C	Dep_self	alcoholfreqweek	108065	-0.002432	0.001161	-0.004707	-0.0001568	-2.095	0.03617	MT-RNR1
Total	26	Affx-92047855	11947	G	Dep_self	alcoholfreqweek	107972	-0.002446	0.001171	-0.004741	-0.0001521	-2.09	0.03663	MT-ND4
Male	26	Affx-79381678	6221	C	Dep_self	smokefreqday	46797	-0.005722	0.00189	-0.009427	-0.002018	-3.028	0.002467	MT-CO1
Male	26	Affx-89025742	6371	T	Dep_self	smokefreqday	46774	-0.005987	0.001987	-0.009882	-0.002092	-3.013	0.002592	MT-CO1
Male	26	Affx-89025745	15218	G	Dep_score	alcoholfreqweek	33646	-0.02676	0.009387	-0.04515	-0.008357	-2.85	0.004372	MT-CYB
Male	26	Affx-34461648	11812	G	Dep_score	alcoholfreqweek	33673	0.01874	0.00664	0.005731	0.03176	2.823	0.004759	MT-ND4
Male	26	Affx-79381667	3992	T	Dep_score	alcoholfreqweek	33666	-0.03425	0.01224	-0.05825	-0.01025	-2.797	0.005154	MT-ND1
Male	26	Affx-34462196	709	A	Dep_score	alcoholfreqweek	33544	0.01319	0.004931	0.003531	0.02286	2.676	0.007453	MT-RNR1
Male	26	Affx-79381683	9667	G	Dep_score	alcoholfreqweek	33668	-0.04237	0.01584	-0.07341	-0.01133	-2.675	0.007473	MT-CO3
Male	26	Affx-79381691	10463	C	Dep_score	alcoholfreqweek	33590	0.01478	0.005774	0.003464	0.0261	2.56	0.01047	MT-TR
Male	26	Affx-34462179	6734	A	Dep_score	smokefreqday	33591	0.04116	0.01684	0.008156	0.07416	2.444	0.01452	MT-CO1
Male	26	Affx-79381716	15928	A	Dep_score	alcoholfreqweek	33503	0.01403	0.00589	0.002484	0.02557	2.382	0.01724	MT-TT
Male	26	Affx-34461806	14905	A	Dep_score	alcoholfreqweek	33643	0.0138	0.005938	0.002165	0.02544	2.325	0.0201	MT-CYB
Male	26	Affx-34462030	4024	G	Dep_score	alcoholfreqweek	33671	-0.03	0.01334	-0.05615	-0.00385	-2.249	0.02455	MT-ND1
Male	26	Affx-89025732	8697	A	Dep_score	alcoholfreqweek	33657	0.01325	0.005962	0.001563	0.02493	2.222	0.02628	MT-ATP6
Male	26	Affx-79381671	4917	G	Dep_score	alcoholfreqweek	33639	0.01308	0.005999	0.00132	0.02483	2.18	0.02926	MT-ND2
Male	26	Affx-79381667	3992	T	Dep_score	smokefreqday	33666	0.02356	0.01081	0.002363	0.04475	2.178	0.02938	MT-ND1
Male	26	Affx-89025778	5147	A	Dep_score	alcoholfreqweek	33655	0.01672	0.007791	0.001449	0.03199	2.146	0.03188	MT-ND2
Male	26	Affx-92047864	9123	A	Dep_score	alcoholfreqweek	33663	-0.02794	0.01324	-0.05388	-0.001994	-2.111	0.03481	MT-ATP6
Male	26	Affx-79381678	6221	C	Dep_score	alcoholfreqweek	33656	0.02872	0.01382	0.001643	0.0558	2.079	0.03763	MT-CO1
Male	26	Affx-79381708	14582	G	Dep_score	alcoholfreqweek	33316	-0.02781	0.01376	-0.05477	-0.0008468	-2.022	0.04323	MT-ND6
Male	26	Affx-89025674	497	T	Dep_self	alcoholfreqweek	46821	-0.002626	0.0009977	-0.004581	-0.0006702	-2.632	0.008498	MT-DLOOP
Male	26	Affx-89025742	6371	T	Dep_self	alcoholfreqweek	46774	0.003958	0.001555	0.0009104	0.007005	2.546	0.01091	MT-CO1
Male	26	Affx-79381672	5004	C	Dep_score	alcoholfreqweek	33675	-0.02569	0.01306	-0.05129	-8.93E-05	-1.967	0.04921	MT-ND2
Male	26	Affx-34461763	13966	G	Dep_self	smokefreqday	46814	-0.00402	0.001634	-0.007222	-0.0008183	-2.461	0.01386	MT-ND5
Male	26	Affx-79381678	6221	C	Dep_self	alcoholfreqweek	46797	0.003402	0.001527	0.0004096	0.006394	2.228	0.02587	MT-CO1
Male	26	Affx-79381667	3992	T	Dep_self	alcoholfreqweek	46814	-0.002965	0.001345	-0.005602	-0.0003289	-2.204	0.0275	MT-ND1
Male	26	Affx-79381691	10463	C	Dep_self	alcoholfreqweek	46701	0.001354	0.0006382	0.0001034	0.002605	2.122	0.03385	MT-TR
Female	26	Affx-86886472	12501	A	Dep_score	alcoholfreqweek	40684	0.04605	0.0138	0.019	0.0731	3.337	0.0008491	MT-ND5
Female	26	Affx-79381658	1719	A	Dep_score	alcoholfreqweek	40671	0.02934	0.008889	0.01192	0.04676	3.301	0.0009658	MT-RNR2
Female	26	Affx-89025745	15218	G	Dep_self	alcoholfreqweek	61106	0.004537	0.001342	0.001907	0.007166	3.381	0.0007221	MT-CYB
Female	26	Affx-79381676	5495	C	Dep_self	alcoholfreqweek	61110	0.006888	0.002149	0.002676	0.0111	3.205	0.001351	MT-ND2
Female	26	Affx-34461684	1243	C	Dep_self	alcoholfreqweek	61206	-0.006482	0.002116	-0.01063	-0.002334	-3.063	0.002193	MT-RNR1
Female	26	Affx-92047855	11947	G	Dep_self	alcoholfreqweek	61157	-0.006501	0.002137	-0.01069	-0.002313	-3.042	0.002348	MT-ND4
Female	26	Affx-89025715	11674	T	Dep_self	alcoholfreqweek	61186	-0.006272	0.002111	-0.01041	-0.002135	-2.971	0.002965	MT-ND4
Female	26	Affx-34462094	5046	A	Dep_self	alcoholfreqweek	61084	-0.006256	0.002108	-0.01039	-0.002125	-2.968	0.002996	MT-ND2
Female	26	Affx-92047849	4529	T	Dep_score	alcoholfreqweek	40725	0.04573	0.015	0.01633	0.07513	3.048	0.002303	MT-ND2
Female	26	Affx-34462179	6734	A	Dep_score	smokefreqday	40611	-0.07695	0.02539	-0.1267	-0.02718	-3.03	0.002444	MT-CO1
Female	26	Affx-79381679	7768	G	Dep_score	alcoholfreqweek	40724	0.03567	0.01206	0.01204	0.0593	2.959	0.003091	MT-CO2
Female	26	Affx-79381704	13780	G	Dep_score	alcoholfreqweek	40701	0.04133	0.01454	0.01282	0.06984	2.842	0.00449	MT-ND5
Female	26	Affx-79381687	10238	C	Dep_score	alcoholfreqweek	40682	0.03917	0.01394	0.01185	0.06649	2.81	0.004952	MT-ND3
Female	26	Affx-79381726	16391	A	Dep_score	alcoholfreqweek	40589	0.04131	0.01471	0.01248	0.07014	2.809	0.004979	MT-DLOOP
Female	26	Affx-79443409	1721	T	Dep_score	alcoholfreqweek	40733	0.04091	0.01465	0.0122	0.06963	2.793	0.005232	MT-RNR2
Female	26	Affx-89025739	3796	G	Dep_score	smokefreqday	40710	0.05329	0.01962	0.01482	0.09175	2.715	0.006623	MT-ND1
Female	26	Affx-79381694	10915	C	Dep_score	smokefreqday	40706	-0.05643	0.02178	-0.09912	-0.01373	-2.59	0.009588	MT-ND4
Female	26	Affx-89025690	15693	C	Dep_score	alcoholfreqweek	40735	-0.04562	0.01825	-0.08139	-0.009847	-2.5	0.01244	MT-CYB
Female	26	Affx-79443438	6047	G	Dep_score	alcoholfreqweek	40715	-0.04472	0.01824	-0.08048	-0.008964	-2.451	0.01424	MT-CO1
Female	26	Affx-92047865	14620	T	Dep_score	alcoholfreqweek	40700	-0.04396	0.01812	-0.07947	-0.008452	-2.426	0.01525	MT-ND6
Female	26	Affx-92047873	11332	T	Dep_score	alcoholfreqweek	40714	-0.04402	0.01821	-0.07972	-0.008328	-2.417	0.01564	MT-ND4
Female	26	Affx-79381709	15043	A	Dep_score	alcoholfreqweek	40643	0.0318	0.01333	0.005673	0.05792	2.386	0.01706	MT-CYB
Female	26	Affx-79443437	5999	C	Dep_score	alcoholfreqweek	40695	-0.04285	0.01849	-0.07909	-0.006612	-2.318	0.02048	MT-CO1
Female	26	Affx-79381715	15924	G	Dep_score	alcoholfreqweek	40592	0.02539	0.01097	0.003885	0.04689	2.314	0.02067	MT-TT
Female	26	Affx-89025749	8994	A	Dep_self	alcoholfreqweek	61168	-0.005155	0.001954	-0.008985	-0.001325	-2.638	0.008347	MT-ATP6
Female	26	Affx-79381710	15257	A	Dep_score	smokefreqday	40713	-0.03704	0.01636	-0.0691	-0.004984	-2.265	0.02354	MT-CYB
Female	26	Affx-34462182	6776	C	Dep_score	alcoholfreqweek	40706	0.02451	0.01131	0.002336	0.04667	2.167	0.03028	MT-CO1
Female	26	Affx-92047859	8448	C	Dep_score	smokefreqday	40728	-0.04387	0.02076	-0.08456	-0.003193	-2.114	0.03454	MT-ATP8
Female	26	Affx-34461957	263	A	Dep_score	alcoholfreqweek	40635	0.04935	0.02477	0.000796	0.09789	1.992	0.04637	MT-DLOOP
Female	26	Affx-79381663	3720	G	Dep_score	alcoholfreqweek	40673	0.04843	0.0244	0.0006085	0.09625	1.985	0.04716	MT-ND1
Female	26	Affx-79381690	10398	G	Dep_score	alcoholfreqweek	40529	0.01251	0.00637	2.87E-05	0.025	1.964	0.04948	MT-ND3
Female	26	Affx-89025711	13617	C	Dep_self	alcoholfreqweek	61130	0.002127	0.0009085	0.0003467	0.003908	2.342	0.0192	MT-ND5
Female	26	Affx-79381660	3197	C	Dep_self	alcoholfreqweek	61082	0.002072	0.0009144	0.0002794	0.003864	2.265	0.02349	MT-RNR2

Note: Dep_self, self-reported depression; Dep_score, depression score

Table S6. Associations of Mitochondrial Gene-by-environment interactions With Self-harm

Gender	Chromosome (CHR)	SNP ID	Base Pair Position (BP)	Allele 1 (A1)	Environment	NMISS	Effect Size (BETA)	Std. Error (SE)	95% CI Lower (L95)	95% CI Upper (U95)	STAT	P-value	Gene
Total	26	Affx-92047859	8448	C	alcoholfreqweek	108702	0.002951	0.001092	0.0008114	0.00509	2.703	0.006868	MT-ATP8
Total	26	Affx-79381672	5004	C	alcoholfreqweek	108661	-0.001785	0.0008598	-0.00347	-9.96E-05	-2.076	0.03792	MT-ND2
Total	26	Affx-79443499	13759	A	alcoholfreqweek	108280	0.001853	0.0009057	7.76E-05	0.003628	2.046	0.04079	MT-ND5
Total	26	Affx-92047864	9123	A	alcoholfreqweek	108623	-0.001741	0.0008621	-0.00343	-5.09E-05	-2.019	0.04349	MT-ATP6
Total	26	Affx-92047856	13020	C	smokefreqday	108655	-0.0022	0.001103	-0.004363	-3.75E-05	-1.994	0.04616	MT-ND5
Total	26	Affx-79381715	15924	G	smokefreqday	108337	-0.001018	0.000519	-0.002035	-1.06E-06	-1.962	0.04976	MT-TT
Male	26	Affx-92047859	8448	C	alcoholfreqweek	47466	0.004072	0.001299	0.001525	0.006619	3.134	0.001726	MT-ATP8
Male	26	Affx-79443532	16362	C	alcoholfreqweek	47337	-0.00123	0.0004961	-0.002203	-0.0002578	-2.48	0.01315	MT-DLOOP
Male	26	Affx-79443499	13759	A	alcoholfreqweek	47275	0.002555	0.001058	0.0004815	0.004628	2.415	0.01573	MT-ND5
Male	26	Affx-79381685	9899	C	smokefreqday	47445	-0.002458	0.001019	-0.004454	-0.0004618	-2.413	0.01581	MT-CO3
Male	26	Affx-89025739	3796	G	smokefreqday	47434	-0.002372	0.001182	-0.004688	-5.59E-05	-2.007	0.04473	MT-ND1
Male	26	Affx-79443531	16356	C	alcoholfreqweek	47240	-0.00149	0.0007451	-0.00295	-2.96E-05	-2	0.04554	MT-DLOOP
Female	26	Affx-34461653	11914	A	smokefreqday	61070	-0.005421	0.001561	-0.008481	-0.002361	-3.472	0.0005169	MT-ND4
Female	26	Affx-34461653	11914	A	alcoholfreqweek	61070	0.004292	0.001524	0.001304	0.00728	2.816	0.004871	MT-ND4
Female	26	Affx-79443409	1721	T	smokefreqday	61242	0.003524	0.001291	0.0009932	0.006055	2.729	0.006353	MT-RNR2
Female	26	Affx-89025778	5147	A	alcoholfreqweek	61210	-0.002378	0.0009245	-0.00419	-0.0005661	-2.572	0.0101	MT-ND2
Female	26	Affx-89025674	497	T	alcoholfreqweek	61218	0.002535	0.001004	0.0005669	0.004504	2.524	0.01159	MT-DLOOP
Female	26	Affx-79443511	14872	T	alcoholfreqweek	61235	-0.005241	0.002147	-0.009449	-0.001032	-2.441	0.01466	MT-CYB
Female	26	Affx-34462196	709	A	alcoholfreqweek	60956	-0.001512	0.0006345	-0.002756	-0.0002682	-2.383	0.01719	MT-RNR1
Female	26	Affx-89025703	16193	T	smokefreqday	61035	0.004116	0.001822	0.0005457	0.007686	2.26	0.02385	MT-DLOOP
Female	26	Affx-34461648	11812	G	alcoholfreqweek	61198	-0.001741	0.0008269	-0.003362	-0.0001204	-2.106	0.03524	MT-ND4
Female	26	Affx-89025751	4793	G	alcoholfreqweek	61195	-0.003231	0.001569	-0.006306	-0.0001558	-2.059	0.03947	MT-ND2
Female	26	Affx-79381691	10463	C	alcoholfreqweek	61090	-0.0015	0.0007363	-0.002943	-5.69E-05	-2.037	0.04163	MT-TR
Female	26	Affx-52321525	150	T	smokefreqday	61136	0.001467	0.0007278	4.04E-05	0.002893	2.016	0.04385	MT-DLOOP
Female	26	Affx-79381671	4917	G	alcoholfreqweek	61164	-0.001492	0.0007563	-0.002975	-1.02E-05	-1.973	0.04846	MT-ND2
